# Osmoregulation capacity in Bulgarian durum wheat

**DOI:** 10.1080/13102818.2014.957054

**Published:** 2014-10-28

**Authors:** Elena Georgieva Todorovska, Violeta Bozhanova, Dechko Dechev, Nelly Valkova

**Affiliations:** ^a^ AgroBioInstitute, Functional Genetics Cereals Group, 8 Dragan Tsankov Street, 1164Sofia, Bulgaria; ^b^ Durum Wheat Breeding and Technology Department, Field Crops Institute, 6200Chirpan, Bulgaria

**Keywords:** durum wheat (*Triticum durum* Desf.), osmoregulation, heritability, SSR markers, principal component analysis (PCA)

## Abstract

The phenotypic variation in osmotic adjustment (OA) capacity of five Bulgarian winter durum wheat genotypes and their progenies was determined using a modified method based on the measurement of seedling growth suppression after three-day exposure to osmotic stress induced by 1 mol/L sucrose. The genetic parameters of the studied trait in a diallel crossing scheme, including the selected genotypes and the microsatellite polymorphism at 43 loci, were determined. The old Bulgarian cultivar Apulicum 233 and all hybrid combinations involving this genotype showed higher OA. In the heritability of osmoregulation ability, the non-additive gene effects (specific combining ability) strongly predominated over the additive ones and had a significant impact on the observed high heterosis effect. Distinct polymorphisms were identified between the studied genotypes. Cluster analysis of the phenotypic data obtained from a multiyear test under water-limited conditions and the molecular data, both based on Euclidean distance, showed similar grouping of the genotypes with specific separation of cultivar Apulicum 233 (high OA) in a single cluster. Principal component analysis revealed not only interrelationships between the important agronomic and morpho-physiological traits in Bulgarian durum wheat under water-limited conditions, but also presence of relations between them and some microsatellite loci located near or within known quantitative trait loci (QTLs) for these traits. Further studies based on segregating population between genotypes with contrasting levels of OA will allow mapping QTLs for phenotypic traits expressed under water deficit and isolation of genes that can be used as potential markers in marker-assisted selection for drought tolerance.

## Introduction

The European Union (EU) is one of the most significant wheat producers worldwide. However, the impacts of the global climate changes in the last decade affect seriously the wheat production and are projected to become more severe. Extreme weather events such as heat waves and dry seasons are expected to become more frequent and intense. Regions most prone to an increase in drought risk are the Mediterranean Basin and vast regions of Central and Eastern Europe. Drought is still a significant challenge facing agricultural researchers and plant breeders, especially in the context of predictions that by the year 2025, about two-thirds of the world's population will live in water-stressed environments and around 1.8 billion people will have to tackle absolute water shortage. The development of cultivars with high-yield stability and high grain and baking quality even under unfavourable environmental conditions is a primary goal in Europe in order for the EU to keep its leading position on the global wheat market.

Durum wheat (*Triticum durum* Desf.) – traditionally a rainfed crop – is often exposed to drought in combination with heat stress, which is the main limiting factor for grain yield.[1–4] The areas in which durum wheat is cultivated in the Mediterranean basin cover a range of macroenvironments with considerably different thermo-pluviometrical regimes.[5,6] Considering the climatic conditions of the Balkan Peninsula, cold and drought are the most damaging abiotic stresses in Bulgaria, which frequently cause substantial losses in crop production and have an unfavourable impact on the economy. The dry years in the recent decade and a half (e.g. 2001, 2003, 2007, 2011) show that this is a rather topical problem for the central and western European countries.

To overcome this problem, it is necessary to develop varieties with higher drought tolerance, on the one hand, and on the other, to improve the efficiency of the existing water-use technologies. A specific hindrance to breeding programmes aimed at creating varieties with stable production under water deficit derives from the fact that it is not possible to carry out effective selection in the years that lack a representative dry period.[7] Another approach to enhance the selection efficiency is to carry out drought tolerance selection on the basis of secondary traits that are easy to measure, highly heritable with low gene–environment (G×E) interaction, genetically correlated with grain yield under stress, and with sufficient genetic variation in the target species.[8] In cereals in particular, there are a number of secondary traits that are generally considered to play a key role in drought tolerance, for example, anatomical features (e.g. root characteristics), physiological traits (e.g. gas exchange, osmotic adjustment), water status indices (e.g. leaf water potential; relative water contents) and cell membrane stability.[9–12] The effectiveness of these secondary traits in selection has been validated in rice,[13,14] wheat [15–17] and maize.[18,19] What is more, some of these traits, e.g. leaf water potential, senescence (stay-green) and osmotic adjustment (OA), have been demonstrated to strongly correlate (positively) with grain yield.[20,21]

OA is a major cellular stress adaptive response in plants that enhances dehydration avoidance and supports yield under stress.[22] Under water deficit, this cellular adaptation mechanism allows plants to actively accumulate solutes, thus resulting in a lower osmotic potential (OP) and the maintenance of high turgor. In wheat, OA capacity is an inherited trait which is controlled by alternative alleles at a major locus on the short arm of chromosome 7A, about 13 cM from the marker.[23–25] Among the indirect methods for estimation of OA, the coleoptile length measurement is an efficient tool to characterize the wheat germplasm for osmotic capacity.

The knowledge of the locations and the effects of genes that influence agronomic traits under drought stress find application in marker-assisted selection. Many of these traits are characterized by quantitative variation and are governed by quantitative trait loci (QTLs), which may interact epistatically and/or with environmental factors. The molecular tools (various molecular markers and mapping populations) necessary to identify the QTLs governing grain yield and yield components [26] as well as G×E interaction are now available for most crops, including wheat.[27–29] The main advantage of marker-assisted selection for traits influenced by drought is that, unlike the conventional breeding methods, it reduces the dependence on specific environmental conditions during the selection process.

In the past decade and a half, microsatellite markers (simple sequence repeats, hereinafter SSRs) have been widely exploited for the construction of wheat maps aligning hundreds of SSRs,[30,31] the Consensus Ta-SSR-2004 map [32] and the Wheat-Composite 2004 map (http://wheat.pw.usda.gov/ggpages/map_summary.html). They have been extensively used for screening genotypes with phenotypic variation in both simple and complex traits related to drought response and for mapping QTLs in wheat grown in water-limited environments.[33,34]

This paper reports phenotypic and genetic variation in the OA capacity of five winter Bulgarian durum wheat genotypes and their progenies. The genetic basis of OA was examined by a diallel crossing scheme and molecular genotyping. The revealed relations between the phenotypic traits expressed under water-limited conditions and some microsatellite loci by principal component analysis (PCA) will allow us to focus further investigations on chromosome regions controlling the expression of these traits, using mapping populations, and thus to further facilitate the selection of genotypes with better performance under unfavourable drought conditions.

## Materials and methods

### Plant material

The study focused on five winter Bulgarian durum wheat (*T. durum* Desf.) cultivars from the Fields Crops Institute – Chirpan, Bulgaria: the old Bulgarian cultivars Аpulicum 233 (A233) and Gergana, the modern ones – Beloslava and Vazhod, and the breeding line D6189.

### Osmotic adjustment capacity of durum wheat cultivars

An indirect method for measurement of seedling growth suppression at osmotic stress [35] was used for all studied genotypes (parents and hybrids). The seeds were sterilized, placed on wet filter paper and turned into rolls. The rolls were placed in plastic boxes containing 100 mL of distilled H_2_O in a thermostat for 48 h, at 26 °C in the dark. After germination, one half of the rolls were left in distillated water (control variant) while the other half was transferred in 1 mol/L sucrose solution, which induces osmotic stress with a pressure of 22 atm, and in this way water deficit was simulated. After three days the length of the roots and shoots was measured in millimetres (mm), in both variants.

The experiment was performed in triplicates for each variant (control and stressed) and genotype. Twenty seedlings per replication were measured. The coefficient of suppression was calculated according to Blum et al. [36]:


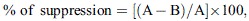


where *A* is the average length of the roots/shoots in the control variant (mm) and *B* is the average length of roots/shoots under osmotic stress (mm).

### Diallel-mating scheme

All five genotypes (A233, Beloslava, Vazhod, Gergana and D6189) were included in diallel crosses, using a scheme based on the Model 1 and Method 4 of Greefing.[37] The experiments were performed during 2006–2009 at the Field Crops Institute, Chirpan, Bulgaria. The parental genotypes were grown to maturity in the greenhouse in pots during three growing seasons (2006–2009) under two water regimes: control condition with normal water supply and moderate drought stress applied at the heading stage (the plants were watered with half of the water amount used for the control variant). The experiment was performed in a randomized block design in triplicate for each genotype and condition. Three plants per genotype were used in each replication. The following phenotypic traits were recorded for each genotype: (1) plant height (PH [cm]); (2) spike length (SL [cm]); (3) peduncle length (PL [cm]); (4) peduncle length/plant height ratio (PL/PH); (5) total tillering (GT); (6) productive tillering (PT); (7) kernel weight per spike (KW/S); (8) number of spikelets per spike (NS/S); (9) kernels number per spike (KN/S); (10) thousand grain weight (TKW [g]).

### Statistical analysis

The results were analysed via a two-way analysis of variance and Duncan's multiple test for significance. Multivariate analysis of the data, including Euclidean distance, clustering of the accessions by the single linkage method and PCA, was used, too. Statistical calculations were carried out by means of a program package Statistica-7 (2004). The general and specific combining abilities (GCA and SCA, respectively) of the hybrids were analysed according to Griffing [37] with the software DIALLEL, kindly provided by the authors Burrow and Coors.[38]

### Microsatellite marker assay

DNA was extracted from leaves of bulked samples (five plants/sample) from each of the genotypes involved in the diallel crossing scheme, using a modified cetyltrimethylammonium bromide (CTAB) method according to Murray and Tompson.[39]

Forty *Xgwm* [30] *Xwmc* [40,41] and *Xbarc* [42] SSR markers were used for genotyping of the selected five durum wheat genotypes. For analysis minimum one marker per chromosome was chosen. Among the markers, three amplified loci on both genomes. The markers were distributed on the chromosomes as follows: 1AS (*Xgwm136*, *Xwmc24*) and 1AL (*Xgwm135*); 1BS (*Xwmc216)* and 1BL (*Xgwm268*, *Xwmc44*); 2AS (*Xgwm95*) and 2AL (*Xwmc170*); 2BS (*Xgwm257*, *Xwmc243*) and 2BL (*Xgwm120*); 3AS (*Xgwm2*) and 3AL (*Xgwm480*); 3BS (*Xgwm285*); 4AS (*Xgwm160*, *Xgwm165*) and 4BL (*Xgwm165*, *Xgwm251*); 5AL (*Xgwm186*, *Xgwm639*); 5BS (*Xgwm234*) and 5BL (*Xgwm408*, *Xgwm639*); 6AS (*Xwmc243*) and 6AL (*Xgwm427*, *Xwmc256*); 6BS (*Xgwm626*) and 6BL (*Xgwm88*, *Xgwm219*); 7AS (*Xwmc479*, *Xwmc158*, *Xgwm60*, *Xgwm233*, *Xgwm260*, *Xwmc603*, *Xbarc108*, *Xwmc83*, *Xwmc9*) and 7AL (*Xwmc273*, *Xgwm282*); 7BS (*Xgwm46*, *Xgwm400*) and 7BL (*Xwmc273*).

Polymerase chain reactions (PCRs) were performed in a final volume of 10 μL in a Perkin–Elmer thermocycler. The reaction mixture contained 250 nmol/L of each primer, 0.2 mmol/L of each deoxynucleoside thriphosphate (dNTP), 1.5 mmol/L of MgCl_2_ and 0.8 U of *Taq* polymerase. Most of the microsatellite loci were amplified with Cy5 labelled forward and unlabelled reverse primers with the exception of *Xgwm6037A* and *Xbarc108-7A*, for which the forward and reverse primers were not labelled. The electrophoresis of PCR products was performed on an automatic sequencer (AFL Express II) or in 2% agarose gels stained with ethidium bromide. Fragment sizes of the labelled PCR products were calculated using Fragment Manager (Pharmacia) software by comparing with internal size standards added to each lane in the loading buffer. The allele lengths were calculated according to the fragment sizes and the number of repeat units at the corresponding locus in Opata or Chinese Spring. In cases where no amplification product was observed, PCR was repeated with newly isolated DNA.

## Results and discussion

Drought stress is one of the major limitations to wheat productivity. It is well known that the conventional breeding strategies have met with little success in breeding for drought tolerance in wheat because of the complex plant response to drought stress that is due mainly to the interaction of different component traits (primary and secondary) with the external environment. Most of the drought related cereal-breeding programmes concentrate on empirical selection of cultivars that give a good yield under drought stress or on physiological selection based on secondary parameters.[8] Among the secondary traits potentially useful for improving plant performance under drought, OA is receiving increased recognition as a major mechanism of drought resistance in crop plants.[43]

### Osmotic adjustment capacity

The OA capacity of Bulgarian durum wheat under water deficit was estimated by an indirect method based on the measurement of coleoptile length. The method of coleoptile growth developed by Morgan [44] is based on the fact that genotypes with better potential for osmoregulation would support more intense cell growth during osmotic stress.

In this study, a modified method based on measurement of seedlings growth suppression after three-day exposure to osmotic stress induced by a 1 mol/L sucrose solution was applied. The analysis showed inhibition of the growth of seedlings in all genotypes included in the experiment. In most of them, the water deficit induced higher level of suppression in the growth of shoots than of the roots. The average values of root and shoot lengths, the ratio between roots and shoots and the suppression coefficient in seedlings as an expression of the capacity of osmotic regulation at the level of whole plants in both the parental genotypes and the hybrid combinations are given in Table 1.

**Table 1.** Tolerance to osmotic stress at seedling stage of five durum wheat genotypes in a diallel cross, averaged for three years.

	Length (cm)						Coefficient of suppression (%)		
	Normal water supply			Osmotic stress					
Genotype	Roots	Shoots	Roots/shoots	Roots	Shoots	Roots/shoots	Roots	Shoots	Seedlings
A233	47.7	50.4	0.9	38.6	34.0	1.1	19.1	32.5	24.7^ab^
Gergana	54.4	49.4	1.1	37.2	23.9	1.6	31.6	52.0	39.7^cde^
Beloslava	54.0	54.7	1.0	41.0	34.8	1.2	24.1	36.4	28.0^abc^
Vazhod	61.4	45.5	1.4	34.1	26.4	1.3	44.5	40.9	36.7^bcde^
D-6189	69.8	58.0	1.2	38.5	34.0	1.1	44.8	41.4	42.1^de^
233 × Gergana	48.9	33.3	1.5	40.3	23.9	1.7	17.6	28.2	22.7^а^
233 × Beloslava	58.7	45.3	1.3	42.9	32.5	1.3	26.9	21.6	24.8^ab^
233 × Vazhod	55.4	45.5	1.1	43.6	29.0	1.5	21.3	36.3	25.4^ab^
233 × 6189	50.8	43.0	1.2	36.9	28.9	1.3	27.4	32.7	28.5^abcd^
Gergana × Beloslava	60.6	50.9	1.2	46.8	31.0	1.5	22.8	39.2	29.9^abcd^
Gergana × Vazhod	56.4	44.2	1.3	32.5	21.2	1.5	42.4	52.0	41.8^de^
Gergana × 6189	48.8	45.6	1.1	30.8	23.0	1.3	38.5	49.6	47.7^e^
Beloslava × Vazhod	60.5	45.3	1.3	39.3	27.9	1.4	35.0	49.9	35.2^bcd^
Beloslava × 6189	48.5	32.6	1.5	29.6	19.8	1.5	38.9	39.3	39.4^cde^
Vazhod × 6189	55.6	38.9	1.4	33.9	21.7	1.6	39.0	44.2	36.6^ bcde^

Note: a, b, c, d values differ significantly at *p* < 0.05.

Among the studied genotypes, the old Bulgarian cv. A233 showed the lowest coefficient of suppression of seedlings (32.5%) and therefore possessed the highest capacity for osmotic regulation. The highest coefficient of suppression (52%) and respectively lowest capacity of osmotic regulation were observed for cv. Gergana. The remaining three genotypes (Beloslava, Vazhod and D6189) showed similar coefficients of suppression and were positioned between the cultivars A233 and Gergana. Among them, cv. Beloslava approximated to A233. All hybrid combinations obtained through the cross between the most tolerant cv. A233 and the remaining four genotypes showed a low coefficient of suppression. The highest suppression coefficient and lower osmoregulation capacity were shown for all F1 progenies of the crosses cv. Gergana × Vazhod, Gergana × 6189, Beloslava × Vazhod and Vazhod × D6189.

As a result of dehydration, the ratio between the length of the roots and the shoots changed, too. In most genotypes these ratios increased during the osmotic stress, but to a different degree, the least in A233 (from 0.9 to 1.1) and to a great extent in cv. Gergana (from 1.1 to 1.6). These data showed that the degree of increase of the root-to-shoot length ratio under osmotic stress can be used as an efficient secondary parameter for OA. The increase of this parameter in drought sensitive genotypes has also been reported by other authors in other species.[45]

In respect to osmotic regulation, the genotypes are usually characterized by the traits OP and relative water content (RWC) in the leaves. These physiological indices are very expensive and time consuming and are not applicable for evaluation of a large number of breeding lines.[46] Therefore, the indirect method applied in our study has a potential to substitute the complicated physiological methods. It can be used for rapid screening of genotypes with better osmoregulation ability and for primary selection of genotypes with better agronomic performance under water deficit in breeding programmes for improvement of drought stress tolerance in durum wheat.

### Genetic parameters

The analysis of variance revealed a statistically significant effect of genotype on the variation of the trait ‘suppression of seedling growth’ under osmotic stress, which is an indication for inheritance of this trait in a broad sense. However, this trait could be effectively used for screening genotypes with high potential for osmoregulation but its applicability in marker-assisted selection in the breeding programmes depends on its inheritance in a narrow sense. The combining ability analysed here gives information about gene actions. The general combining ability effects are consequences of the additive gene actions, while the effects of the specific combining ability reflect only the non-additive ones.

The diallel analysis of the examined traits used as an indirect index for osmoregulation ability showed the significance of the effects of both general and specific combining ability over the three-year period of the study (Table 2). This means that the inheritance of the higher levels of osmoregulation was determined by both the additive and non-additive gene effects. Moreover, the non-additive gene effects (SCA) strongly predominated over the additive ones. The observed high heterosis effect leading to a decrease in the suppression coefficient, i.e. to higher level of osmoregulation, was due basically to non-additive gene effects. Among the genotypes included in the investigation, cv. A233 was proved as a positive common combiner for the trait high osmotic regulation, while cv. Gergana, as a negative one.

**Table 2.** Significance of GCA and SCA and the ratio between GCA and SCA variances.

Suppression coefficient	General combining ability (GCA)	Specific combining ability (SCA)	GCA/SCA
roots	***	***	≤1
shoots	***	***	≤1

Note: ***p ≤ 0.1%.

The information about the heritability of indirect physiological traits is important for development of adequate breeding strategies for improvement of drought resistance, having in mind the low inheritance of the yield in different environments due to large interaction between the genotypes and environments (G×E).[21] The results from the diallel analysis of the trait ‘suppression of seedling growth’ at osmotic stress revealed the significance of variances of both GCA and SCA. This is an indication that the selection of single plants with variation in the investigated trait has to be shifted in the later segregating progenies. Then the efficiency of selection will be vastly higher due to decrease of SCA effects as a result of the natural increase of homozygosity in every subsequent generation of self-pollination. The prevalence of the non-additive gene effects observed in our investigation supports the findings of many authors in other cereals that a relative little number of loci are responsible for the variability in this trait.[24,47,48]

### Molecular marker screening

In addition to physiological parameters and agronomical observations, the genomics-based approaches provide excellent opportunities to search for loci that correlate with the observed phenotype variation and to map QTLs for drought tolerance (reviewed in [49]). Molecular markers have been extensively used for characterization and selection of wheat germplasm with better agronomic performance. The discovery of markers associated with drought tolerance phenotypes is an important step in identification and isolation of genes controlling these specific responses.

In our study, 40 SSR markers were used to determine the genetic differences between five Bulgarian durum wheat genotypes showing different capacity for osmotic regulation as determined on the basis of suppression of seedling growth under water deficit induced by osmotics. The microsatellite markers amplified a total of 43 loci on the A and B genomes of all seven chromosomes of durum wheat. They detected a total of 110 alleles, with a mean of 2.683. Of the loci tested here, the most polymorphic ones were *Xgwm136-1AS* (six alleles; with a polymorphism information content (PIC) of 0.772), *Xwmc273-7A* (five alleles; PIC = 0.745) and *Xgwm 2-3A*, *Xgwm88-6B*, *Xgwm95-2A*, *Xgwm120-2B*, *Xgwm268-1B*, *Xgwm408-5B*, *Xgwm639-5B* with four alleles and *PIC* values ranging from 0.53 to 0.67. Six of the tested loci were monomorphic. The mean genetic diversity in the whole set of the tested durum wheat genotypes was 0.386, while the estimated genetic diversity among the genotypes was higher between A233 and cv. Beloslava, which is the most closely related cultivar as regards the traits specific for osmotic regulation. The lowest diversity was observed between the tolerant A233 and the sensitive cv. Gergana, with a value of 0.534. A total of 27 polymorphisms were found between them across the 43 tested SSR loci. The highest level of polymorphism was observed on chromosome 7A on both the short arm (*Xgwm233*, *Xwmc479*, *Xgwm260*, *Xbarc108*, *Xwmc603*) and the long one (*Xwmc9*, *Xgwm282*, *Xwmc273*), where QTLs for different agronomic, physiological traits and yield, metabolites and osmotic resistance have been previously mapped.[25,50–53]

### Relation between the phenotypic traits and microsatellite loci

**Figure 1.**
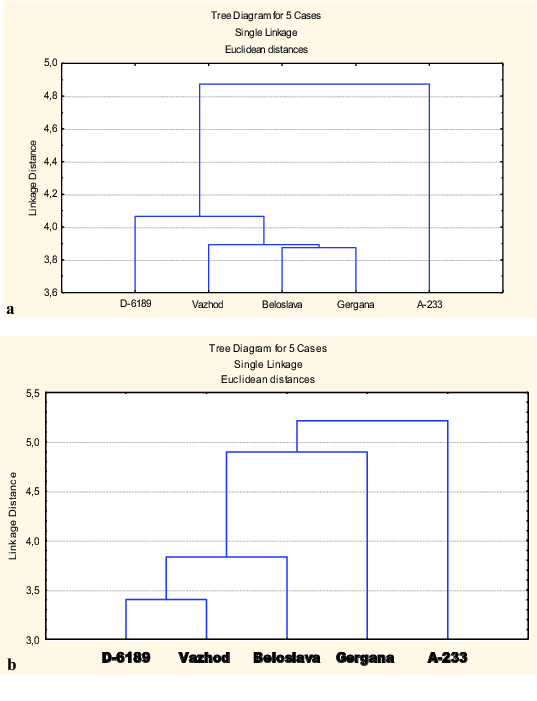
Dendrogram of five durum wheat genotypes based on phenotypic traits (means of three years) under water stress conditions (a) and on microsatellite markers (b).

Two separate cluster analyses based on phenotypic and molecular data were performed to categorize the studied durum wheat genotypes. The dendrogram constructed on the phenotypic traits related to yield components and OA capacity under water deficit (Figure 1(a)) classified the genotypes into three groups. The most distant genotype of the studied ones was the old Bulgarian cv. A233 showing the highest osmoregulation ability. A second group consisting of only one genotype, the breeding line 6189, was separated at 81% after the first segregation. The cultivars Beloslava, Gergana and Vazhod grouped in the third cluster, which illustrates their closeness.

The cluster analysis based on the genotypic data obtained through genotyping using 43 SSR markers (Figure 1(b)) showed a similar grouping pattern of the cultivars. Both analyses revealed that the genotype with the high osmoregulation ability (cv. A233) has a specific location, in a separate and more distant group. The observed similarity between both dendrograms is an evidence for the presence of a relation, to some extent, between the phenotypic data for OA and yield-related traits under the conditions of limited water supply and the molecular data.

PCA was performed for visualization of the relations between the phenotypic traits, on one hand, and between them and microsatellite loci, on the other. Recently, some authors begin to use multifactorial analyses for searching of relation between phenotypic traits and molecular data.[54–57] Yan and Tinker [55] showed that biplot analysis can be a useful tool for investigations related to marker-based selection in different environments.

**Figure 2.**
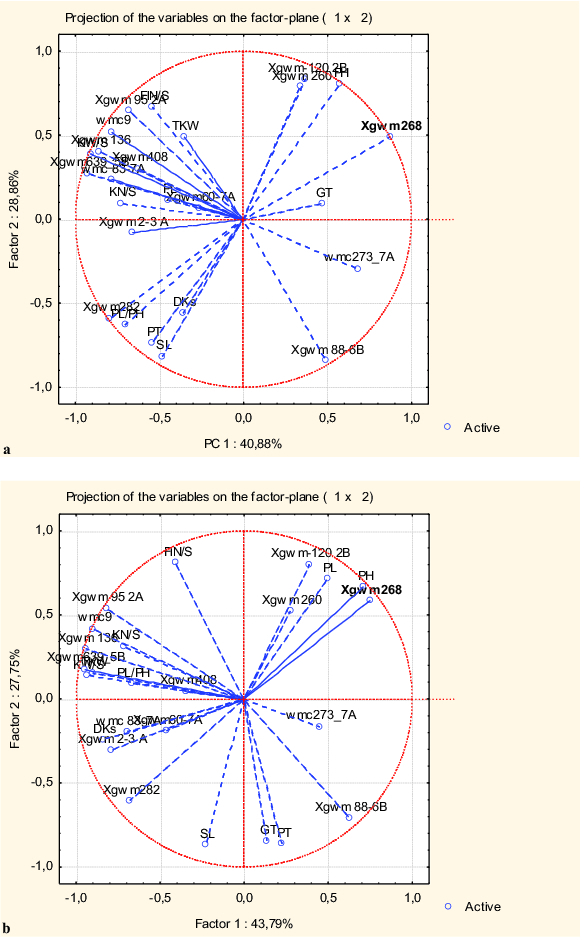
PCA of phenotypic traits and molecular markers in five Bulgarian durum wheat genotypes grown in a greenhouse during 2006–2009 at water-limited conditions (a) and well-watered conditions (b).

Graphic expression of the analysis of the studied genotypes grown during three seasons in two water regimes under greenhouse conditions is shown in Figure 2(a) (water deficit) and 2(b) (control conditions). The first two PCs accounted for 70% (40.88% and 28.86% by PC1 and PC2, respectively) of the total variation observed among durum wheat genotypes at water deficit, and for 72% (43.8% and 27.8% by PC1 and PC2, respectively) for well-watered conditions. Almost all phenotypic traits and the microsatellite loci studied here were presented with long vectors revealing that they have large contribution to the total observed variation. According to Yan et al.,[58] the correlation coefficient between the traits depends on the cosine of the angle between the vectors of any two traits. However, the cosine of the angles does not translate into correlation coefficient, since PC1 and PC2 do not explain all variation in the dataset. The trait vectors that are approximately at a right angle are not closely related, while the oppositely oriented, at a 180° angle, correlate negatively.

The results of the PCAs presented in Figure 2(a) and 2(b) revealed both positive and negative relations between the studied phenotypic traits and between the traits and SSR loci used. Only few correlations between the phenotypic traits remained the same under the applied distinct regimes. The correlations between the traits related to spike productivity (KN/S, KW/S, TKW) and between DKs and PL/PH were positive, while those between SL and PH, as well between PH and PT were negative. The correlations between the remaining traits changed under water-stress conditions. For example, the trait peduncle length (PL) correlated positively only with plant height (PH) under well-watered conditions. However, under water deficit it correlated with the traits related to spike productivity such as FIN/S, KN/S, KW/S and TKW. The trait PL/PH correlated with KW/S, KN/S, TKW and DKs at normal conditions, while under stress, with the other two traits, PT and SL.

Alterations in relations between some phenotypic traits and microsatellite loci at different water regimes were also found. The following identical relations were observed under both conditions: positive ones between GT and *Xgwm268* (1BL); PH and loci on 1BL (*Xgwm268*), 2BL (*Xgwm120*) and 7AS (*Xgwm260*); SL and *Xgwm282* (7AL); KN/S, KW/S and TKW with these on chromosomes 3AS (*Xgwm2*), 5BL (*Xgwm639*) and 7AS (*Xgwm60*, *Xwmc83*); FIN/S and loci on 2AS (*Xgwm95*) and 5BL (*Xgwm408*); and negative ones, between FIN/S and *Xgwm88* (6B).

The remaining traits were in relation with different sets of chromosome loci in each separate environment. Under water deficit, strong positive relations were observed between PL and loci on chromosomes 7AS (*Xwmc83*, *Xgwm60*), 5BL (*Xgwm639*, *Xgwm408*) and 1AS (*Xgwm136*); PL/PH and *Xgwm282* (7AL); TKW and FIN/S with these on 2AS (*Xgwm95*) and 7AS (*Xwmc9*). Negative relations were found between the traits SL, PT, PL/PH and loci on chromosomes 2BL (*Xgwm120*) and 7AS (*Xgwm260*); and between DKs and these on chromosomes 7AS (*Xgwm260*) and 2BL (*Xgwm120*). Under control conditions, different relations were identified: positive ones between PL and *Xgwm268* (1BL), *Xgwm260* (7AS), *Xgwm120* (2BL); PL/PH and *Xgwm408* (5BL), *Xgwm639* (5BL), *Xgwm136* (1AS), *Xwmc9* (7AS), *Xgwm95* (2AL); GT and PT and *Xgwm88* (6BL); and negative ones between PH and *Xgwm 282* (7AL) and between GT and PT and *Xgwm95* (2AS).

The interpretation of the PCA data allows some significant relations to be outlined among the phenotypic traits studied here and between them and some microsatellite loci. Figure 2(a) shows the presence of correlations between three morphological traits related to productivity under drought stress. The PL correlated positively with all traits related to spike productivity as FIN/S, KN/S, KW/S and TKW, while the ratio PL/PH correlated with PT and SL. There is abundant evidence that the trait PL is a useful indicator of yield capacity in dry environments. Kaya et al. [59] observed a strong positive correlation between PL and grain yield. Several reports confirmed the important role of PL in the storage and transfer of carbohydrate resources for increasing grain productivity under drought stress.[60–62] In our study, under water-limited conditions strong positive relations between PL and loci on chromosomes 7AS (*Xwmc83*, *Xgwm60*), 5BL (*Xgwm639*, *Xgwm408*) and 1AS (*Xgwm136*) were observed. These observations are not surprising because various QTLs have been reported in wheat, mainly based on experiments with an objective to unravel the genetic basis of grain yield and morpho-physiological traits related to yield under stressed and non-stressed conditions. Quarrie et al. [50] have mapped QTLs for drought resistance in hexaploid wheat on chromosomes 1A, 1B, 2A, 2B, 2D, 3D, 5A, 5B, 7A and 7B, of which those on 7A are located in the regions covering the polymorphisms observed by us. A 7A locus that shows increased spike length, higher grain number, and increased yield, particularly under severe drought stress, has been also identified.[63] Recently, Hill et al. [52] using mapping population in hexaploid wheat identified a 1.0 cM region between *Xgwm60* and *Xwmc283* on chromosome 7AS, affecting several organic acids, the amino acid Gln and a range of agronomic traits, including glaucousness, PL, grains per square meter, grain yield, and harvest index under drought stress conditions. The QTL for grain yield, Glc-6-P (in the 1.1 cM interval between *Xwmc83* and STM0511TCTG) and sodium exclusion in this region was also reported by Edwards et al.[64] The sodium-exclusion QTL is associated with osmoregulation capacity at salt stress conditions.[65] A new putative QTL on chromosome 7A associated with the candidate gene 6-sucrose: fructan fructosyltransferase, which might be implicated in OA during drought stress through the accumulation of fructan, is also mapped.[51]

A relation between DKs and PH under water deficit was found in this study. Many authors have reported a positive correlation between PH and yield under drought stress in wheat.[66–68] Due to the capacity of tall wheat genotypes for better water extraction from soil and the effective role of stored materials in the stem of these genotypes, they perform better as compared to short genotypes.[69] Among all genotypes used in this investigation, cv. A233 (highest OA) was the one with the longest stem. Furthermore, a relation between PH and two loci, *Xgwm260* and *Xgwm120*, on chromosomes 7AS and 2BL, respectively, was detected in both environments studied by us. Maccaferri et al. [27] also identified a major QTL for PH on chromosome 7AS (proximal region, QPht.idw-7AS) in durum wheat, which is stably expressed across six experimental environments. A relation between PH and the marker *Xgwm268* (1BL) was also observed in both environments tested here. This locus is positioned to the QTLs for PH reported by Maccaferri et al. [28] and Laido et al.[70]

Several authors have noted that the trait thousand kernel weight (TKW) can serve as an important selection criterion for high-yielding durum wheat genotypes under water-deficit conditions. Maccaferri et al. [28] identified that TKW correlates positively with grain yield, particularly in medium- and low-productive environments. In the study of Royo et al.,[71] TKW has been found as a trait most related to yield, since it explained 63% of yield variations under Mediterranean agro ecosystems. According to the data reported by Nouri et al.,[72] durum wheat genotypes with higher yield under drought conditions are characterized with higher TKW and PH. In our study, TKW correlated strongly with KW/S, PH/PL and KN/S in non-stressed conditions and was found to be in relation predominantly with loci on 5BL chromosome (*Xgwm639* and *Xgwm408*), where a QTL for this trait was found in the studies of Laido et al. [70], Reif et al. [73] and Peleg et al. [74] (Figure 2(b)). However, in the stressed conditions, TKW correlated more strongly with FIN/S and showed a relation with loci on 2AS (*Xgwm95*) and 7AS (region *Xgwm60*-*Xwmc9*) (Figure 2(a)), where QTLs for this trait [28,70] and heading date [70,75] have been reported. In addition, a relation between TKW and locus *Xgwm120* on 2BL, although not as strong, was also observed, which corresponds to the TKW-QTL mapped by Laido et al. [69]. The experimental data presented here showed that cultivar A233 is characterized not only with the highest PH, but also with increased TKW under water-deficit conditions in comparison to all remaining genotypes.

A relation between the lowest DKs (highest OA) and *Xgwm260* (7AS) was also identified (Figure 2(a)). According to the Consensus Ta-SSR-2004 map, this locus is situated near to the QTL for membrane stability under water stress in hexaploid wheat positioned between the loci *Xwmc603* and *Xbarc108* on 7AS.[53]

Although PCA revealed the interrelationships between the traits, it also provides an abstract visualization of the relations between some SSR loci and agronomical, morphological and physiological traits related to productivity under water-deficit conditions. Most of the observed relations are in agreement with the QTLs already identified by the use of multiyear tests of large number of segregating populations and association mapping populations in a broad range of environments.

## Conclusions

In this study, suppression of seedling growth induced by osmotics (as a secondary physiological trait) was proven as useful tool for distinguishing of winter durum wheat genotypes with different levels of tolerance to drought. The high heritability of the trait OA, the determined gene effects and the high heterosis effect will permit an adequate breeding strategy for improvement of the target traits. A large number of polymorphisms between the highly tolerant cv. Apulicum233 and the sensitive cv. Gergana with predominant location on chromosome 7A were observed. PCA revealed the presence of relations between some microsatellite loci and agronomical, morpho-physiological traits related to productivity in durum wheat under water-stressed conditions. These results hold out further opportunities for the development of genomic tools for mapping QTLs, using segregation population and isolation of important genes responsible for drought stress tolerance in Bulgarian winter wheat.
